# Chemical and Enzymatic Probing of Viral RNAs: From Infancy to Maturity and Beyond

**DOI:** 10.3390/v13101894

**Published:** 2021-09-22

**Authors:** Orian Gilmer, Erwan Quignon, Anne-Caroline Jousset, Jean-Christophe Paillart, Roland Marquet, Valérie Vivet-Boudou

**Affiliations:** Université de Strasbourg, CNRS, Architecture et Réactivité de l’ARN, UPR9002, F-67000 Strasbourg, France; o.gilmer@ibmc-cnrs.unistra.fr (O.G.); e.quignon@ibmc-cnrs.unistra.fr (E.Q.); ac.jousset@ibmc-cnrs.unistra.fr (A.-C.J.); jc.paillart@ibmc-cnrs.unistra.fr (J.-C.P.)

**Keywords:** RNA, structure, enzymatic probe, chemical probe, SHAPE, capillary electrophoresis, high-throughput sequencing, mutational profiling

## Abstract

RNA molecules are key players in a variety of biological events, and this is particularly true for viral RNAs. To better understand the replication of those pathogens and try to block them, special attention has been paid to the structure of their RNAs. Methods to probe RNA structures have been developed since the 1960s; even if they have evolved over the years, they are still in use today and provide useful information on the folding of RNA molecules, including viral RNAs. The aim of this review is to offer a historical perspective on the structural probing methods used to decipher RNA structures before the development of the selective 2′-hydroxyl acylation analyzed by primer extension (SHAPE) methodology and to show how they have influenced the current probing techniques. Actually, these technological breakthroughs, which involved advanced detection methods, were made possible thanks to the development of next-generation sequencing (NGS) but also to the previous works accumulated in the field of structural RNA biology. Finally, we will also discuss how high-throughput SHAPE (hSHAPE) paved the way for the development of sophisticated RNA structural techniques.

## 1. Introduction

RNA molecules are key players in a variety of biological events, and this is particularly true for viral RNAs. It has been known since 1949 [1] that the infectivity of some viruses is driven by viral RNA (vRNA), and that vRNA alone can infect plants [2]. Therefore, to better understand the replication of those pathogens and try to block this process, special attention has been paid to the structure of their RNAs. Indeed, many functions that are essential to viruses are encoded by specific RNA domains that are engaged in long-range interactions or promote specific interactions with metal ions, proteins, or other RNAs. Additionally, vRNAs can be replicated, spliced, translated, or packaged as genomic RNAs (gRNAs) into new virions. Because these processes are highly dependent on interactions enabled or prevented by RNA structure, full understanding of the molecular mechanisms involved requires characterization of these RNA structures. However, RNAs are dynamic molecules that can fold into various conformations, depending on environmental conditions. Their flexibility and large size often make them unsuitable for study by the most powerful structural approaches, such as X-ray crystallography [3,4], nuclear magnetic resonance, [5] or cryo-electron microscopy [6], although this situation is changing in recent years thanks to the coupling of cryo-EM with high-throughput biochemistry and computer modeling of the 3D structure [7]. Nevertheless, by the second half of the 20th century, methods to probe the structure of RNA in solution were developed; even if these techniques have evolved over the years, they are still in use today and provide useful information on the folding of RNA molecules, including vRNAs.

The aim of this review is to offer a historical perspective on the structural probing methods used to decipher RNA structures before the development of the high-throughput selective 2′-hydroxyl acylation analyzed via the primer extension (hSHAPE) methodology, discussing examples taken from the world of viruses and how they have influenced the probing techniques since 2005. Indeed, nowadays, we have numerous sophisticated tools to assess RNA structure in vitro, in cellula, and even in viro, either alone or in complex with various macromolecules. These technological breakthroughs using enzymatic and chemical probes, which are detected through advanced methods [8], were made possible thanks to next-generation sequencing (NGS) approaches but also to the previous works accumulated in the field of structural RNA biology [9]. Therefore, to understand where most of the currently used methods originated from, it is necessary to go back to the “Pre-SHAPE era” of RNA structural biology. Finally, we will also discuss how hSHAPE paved the way for the development of sophisticated RNA structural techniques that provide more detailed structural information. However, the crosslinking-based structural methods, which have also been developed recently and are complementary to the RNA probing methods, are not reviewed here (for recent reviews of these techniques, see [10,11,12]).

## 2. History of the RNA Structure Epic: From Scratch to SHAPE 

Since the first mention of RNA, referred to as “yeast nucleic acid” in the early 1900s, numerous key discoveries have been made concerning its structure. In 1954, the hypothesis that RNA is a “2′-3′-5′ branched” molecule was abandoned in favor of the linear 5′-3′ chain [13]. In 1956, Rich and Davies [14] showed that RNA is able to form double-stranded structures, in a “DNA-like” fashion. The next year, Felsenfeld et al. [15] uncovered the complexity of the RNA structure, with a triple-stranded RNA structure observed by combining X-ray diffraction and sedimentation studies. From that point onwards, special attention was brought to RNA structure and the methods to determine it.

### 2.1. Birth of RNA Structure Probing

Long before the first observation of a structured RNA molecule (by Rich and Davis in 1956), ribonucleases were known to degrade ribonucleic acids [16]. However, little was known about their specificity towards nucleotide identity or RNA structures. An early report demonstrating the nucleotide specificity of ribonucleases was published in 1957, albeit with some errors, when RNase T1 and RNase T2 were purified from *Aspergillus oryzae* by Sato and Egami [17]. Then, in 1962, it was hypothesized that double-stranded RNA is resistant to nucleases [18,19,20]. Finally, in 1965, Holley et al. [21] took advantage of the properties of two RNases, RNase T1 (specific cleavage of RNA at the 3′ phosphate of unpaired Gs) [22] and pancreatic RNase (specific cleavage at the 3′ of unpaired Us and Cs [23]), to study the structure of yeast tRNA^Ala^. Comparing the short and long fragments obtained under complete and partial digestion, respectively, they were able to identify and arrange the different fragments, in relation to each other. They proposed three structural models for tRNA^Ala^, including the now classical cloverleaf structure.

In parallel to the structural studies with enzymes, chemical reagents that are able to interact or modify nucleic acids were also investigated, in order to understand the properties of RNA. Historically, one of the first chemicals known for its ability to interact with nucleic acids was 1-chloro-2-[(2-chloroethyl)sulfanyl]ethane, also known as mustard gas [24]. This compound, as well as other alkylating agents, was shown to efficiently inactivate viruses, such as the tobacco mosaïc virus [25]. Some years later, methylation of adenosine, cytosine, and guanosine, by the nowadays widely used dimethylsulfate (DMS), was demonstrated by Lawley and Brookes [26]. In the same period, Gilham described, for the first-time, a carbodiimide chemical reagent able to modify uridine and guanosine, thus rendering these nucleotides less sensitive to RNase cleavage [27]. In 1965, the link with the secondary structure of nucleic acids was made indirectly by using the property of carbodiimide derivatives to protect the U and G nucleotides from specific RNase digestion [28,29] and not yet as a direct chemical probe by itself, as it is now used.

### 2.2. Childhood of RNA Probing and Investigation of Viral RNA Structures

#### 2.2.1. Characteristics and Specificities of the Pre-SHAPE Probes

**Figure 1:**
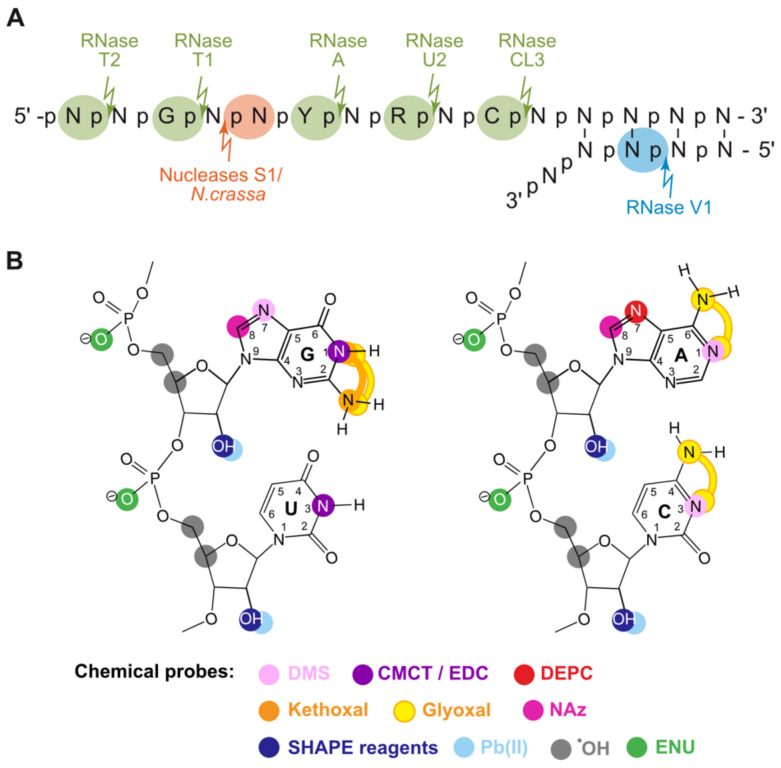
Enzymatic and chemical probes available nowadays. (**A**) Common enzymatic probes and their targets on ss- and dsRNA. The arrows and the highlighted nucleotides indicate whether the fragment formed after strand scission is 3′ or 5′ phosphate. (**B**) Main chemical probes and their target positions on base, sugar, and phosphate. DMS: dimethylsulfate; CMCT: 1-cyclohexyl-3-(2-morpholinoethyl) carbodiimide metho-p-toluenesulfonate; EDC: 1-ethyl-3-(3-dimethylaminopropyl)carbodiimide; DEPC: diethylpyrocarbonate; kethoxal: 3-ethoxy-1,1-dihydroxy-2-butanone; SHAPE reagents are NMIA: N-methylisatoic anhydride, BzCN: benzoylcyanide, and NAI: 2-methyl nicotinic acid imidazolide; FAI: 2-methyl-3-furoic acid imidazolide; 1M6: 1-methyl-6-nitroisatoic anhydride; 1M7: 1-methyl-7-nitroisatoic anhydride and 2A3: 2-aminopyridine-3-carboxylic acid imidazolide; NAz: nicotinoyl-azide; Glyoxal: ethanedial [40]; ENU: ethyl-nitrosourea; Pb(II): lead ion; ^•^OH: hydroxyl radical.

From 1965 to 2005, 40 years of RNA probing research has led to the development of a wide range of tools available to scientists (Figure 1 and Table 1).

The most commonly used RNases specifically cleave single-stranded nucleotides. Some of them cleave RNA molecules without sequence specificity, while others have a more or less pronounced preference for one or several nucleotides. For example, *Neurospora crassa* endonuclease [30], RNase T2 [17], and nuclease S1 from *Aspergillus oryzae* [31] cleave single-stranded RNA (ssRNA), with limited dependency on the nucleobase identity. RNase T2, together with RNase T1 (see below), presents the additional advantage of being active at neutral and basic pH, even in the absence of divalent cations and is, thus, ideal for studying the effects of pH and Mg^2+^ ions on the RNA secondary structure [32]. RNase T1 from *Aspergillus oryzae* specifically cleaves RNA at the 3′ phosphate of unpaired G’s [22], and chicken liver RNase 3 (CL3) cleaves ssRNA at Cs and, to a lesser extent, at As and Us [33]. RNase U2, from *Ustilago sphaerogena*, cleaves the 3′-5′ phosphodiester bonds of unpaired As and Gs [34], and RNase A cuts after unpaired pyrimidine residues, with a preference for Cs and especially CpA motifs [35]. In contrast to these ssRNA-specific enzymes, RNase V1, also known as cobra venom RNase (since it was isolated from the venom of the Naja snake *Naja oxiana* [36,37]), selectively cleaves double-stranded RNA (dsRNA), or staked nucleotides, by generating 5′-phosphate ends [38]. It has to be noted that nowadays, RNase V1 is still one of the very few probes that gives a positive signal for the ds regions, together with hydroxyl radicals generated from the intercalating agent MPE-Fe(II). The activity and optimal conditions of use for many nucleases are detailed in Desai et al. [39]. The main limitations of RNases as structural probes come from their size, which makes them sensitive to steric hindrance and prevents their use in cells or viral particles. Furthermore, if the reaction conditions are too strong, secondary cleavages, that do not reflect the native RNA secondary structure, may occur.

Beside enzymatic probes, chemical probes were also developed and have several advantages. Indeed, they are smaller in size, potentially effective in vivo, compatible with most buffer components, and active over a wide range of pH, salt, and temperature. Most of them target ssRNAs and show some base specificity. Kethoxal, which was originally known as an antiviral agent (thanks to its ability to react with Gs [41]), was used to probe N1- and NH_2_-2-G of the ssRNA regions [42]. In 1970, Öberg et al. showed that diethylpyrocarbonate (DEPC) modifies ssRNA, but not dsRNA, by conducting studies on polioviruses [43]. It was not until a year later that DEPC was found to react with N7-A [44]; in 1980, Peattie and Gilbert [45] described a procedure to probe stacked adenosines by treating RNA with DEPC and then inducing a cleavage with aniline. Additionally, 1-cyclohexyl-3-(2-morpholinoethyl) carbodiimide metha-p-toluene sulfonate (CMCT) targets N3-Us and N1-Gs [46], whereas DMS modifies N3 and N1 of unpaired Cs and As, respectively, and the N7 of Gs was not involved in tertiary interactions [45]. Interestingly, methylation of the N1-A position was only discovered in 1985, since it can be detected solely with the primer extension readout method (see below) [47]. The modification of the Hoogsteen face of Gs is interesting, since it allows for the use of DMS to probe G-quadruplexes [48]. Other chemicals react with the sugar-phosphate backbone and, therefore, do not depend on the nature of the base. Such compounds include ethyl-nitrosourea (EtNu or ENU), which alkylates the oxygens of the phosphate groups of ss- and ds nucleic acids that are not engaged in tertiary interactions or in cation coordination. After an additional alkaline treatment, the phosphotriester is hydrolyzed, resulting in RNA strand scission [49,50]. Lead (II) cleaves the phosphate backbone of ssRNAs, with pronounced cleavage in metal ion binding pockets; the highly reactive hydroxyl radicals probe the solvent accessibility of the nucleic acid backbone when generated from Fe(II) coordinated to soluble EDTA [51] or double-stranded structures when generated from Fe(II) in complex with EDTA linked to the intercalator methidiumpropyl (MPE-Fe) [52].

In the first experiments in the 1960s, detection of the modified nucleotides was achieved by paper or gel electrophoresis of the small fragments obtained after the successive digestion of RNAs and the laborious work of puzzle reconstruction [21,53]. From the 1970s, the detection of modification or cleavage sites was achieved by one of two methods, both of which involve ^32^P (or sometimes ^35^S) labeling. For probing experiments of small RNAs (or of the extremities of long RNAs), direct detection of strand scissions of 3′ or 5′ end-labeled RNAs is the method of choice. In that case, the cleavage of the ribo-phosphate backbone can, of course, be achieved by enzymatic probes or chemical probes, such as lead (II) or hydroxyl radicals. However, other chemical reagents that, under normal conditions, only modify RNA can be combined with a subsequent treatment that induces strand scission at the modification site. For instance, DMS modified guanosines, when reduced by NaBH_4_, then treated with aniline, results in strand cleavage [54]; whereas N3 alkylated cytosines need to be treated with hydrazine, and then with aniline, for strand scission [55]. It has to be noted that treatment with hydrazine is not specific, since it may induce cleavage at the U positions, although these nucleotides are not probed by DMS. DEPC is also compatible with this detection method when coupled with aniline treatment [56], as well as ENU, since the phosphotriester formed after ENU treatment are easily hydrolyzed under mild alkaline conditions [57]. The RNA fragments are separated by polyacrylamide gel electrophoresis and by comparison with a sequencing ladder; thus, the positions of the cleavages in the RNA molecule are identified.

An alternative method emerged when Qu et al. [77] and Moazed et al. [78] found that it was possible to map the location of enzyme cleavage sites or chemical modification at Watson–Crick positions (kethoxal, DMS, and CMCT) with reverse transcription stops. This technique involves the extension of a radiolabeled primer with reverse transcriptase (RT) and the subsequent resolution of the cDNA population, by denaturing polyacrylamide gel electrophoresis and detection, either by autoradiography or phosphorimaging.

#### 2.2.2. Early Applications to Viral RNAs

The biochemical probing techniques mentioned above were often developed to solve eukaryotic and prokaryotic RNA structures, but they were also used alone or in combination and proved their efficiency in the structure determination of viral RNAs transcribed in vitro (extracted from virions or directly inside the particles) (Table 1). In the following sections, we describe some examples of the early applications of RNA probing for the elucidation of vRNA structures.

##### 3′-Terminal Region of Plant Viruses: tRNA Mimicry for Replication

Since the first RNA structures ever identified were tRNAs, it was not surprising that the first recognized viral structures were tRNA-like structures present in the 3′ termini of ssRNA plant viruses (3′TLS). The specific features of some plant viral genomes were discovered after the observation of a linkage between the terminal adenosine of the turnip yellow mosaic virus (TYMV) RNA and a valine amino acid [79]. Structural and functional studies were performed to try to understand why vRNA was aminoacylated. In the early 1980s, Ahlquist et al. [63] studied the RNA 3′-terminal region of different bromoviruses and a cucumovirus. Using S1 nuclease to probe the RNA secondary structure, they showed that the 3′-terminal region was highly conserved, suggesting a strong functional constraint, even though the TLS secondary structures were quite different from that of canonical tRNAs. Nevertheless, the cellular aminoacyl-tRNA synthetases were able to recognize the 3′TLS, in order to aminoacylate the terminal CCA_3′OH_ at levels similar to those of the cellular tRNA molecules.

Following these finding, many studies were performed on the 3′TLS from TYMV. Florentz et al. used ENU to probe the RNA structure, and they complemented that tool with a combination of enzymes, such as nuclease S1, RNase CL3, T1, and V1 [60]. In the meantime, Rietveld et al. [61] proposed a secondary structure for the TYMV 3′ terminal region. They used RNase T1, V1, and nuclease S1, as well as the chemical probes DMS and DEPC. At that time, DMS had already been used to decipher the global ss or ds behavior of an RNA structure [80] and to study the structure of yeast tRNA [46]. Rietveld et al. demonstrated the existence of a pseudoknot, which was absolutely necessary for the aminoacylation of the 3′TLS. Furthermore, they showed that the 3′-end of TYMV gRNA does not present the conventional acceptor stem of tRNA, but they proposed a tertiary structure, which nevertheless resembled the acceptor arm of tRNA. This arrangement is conserved in other plant viruses of the tymovirus and tobamovirus groups. In 1983, the probing of cucumovirus and bromovirus gRNA 3′-end with RNase T1 was performed by Joshi et al. [81]. These authors studied the minimal sequence necessary to form TLS, as well as the implication of Mg^2+^ ions into aminoacylation. Up to now, the exact function of the 3′TLS remains unclear [82]. Yet, numerous insights on 3′TLS were acquired. Three different classes of 3′TLS have been identified, characterized by the amino acid bound to their 3′CCA_OH_, with three different types of secondary structures giving rise to very similar 3D structures [83].

##### 3′-Terminal Region of Plant Viruses: Cap Independent Translation Enhancers

In ssRNA plant viruses, TLS are not the only structure found in the 3′terminal region. Some viruses devoid of 5′ cap and 3′ polyA tail use a non-canonical mechanism to recruit ribosomes to their 5′-end, by a specific RNA element [84]. These motifs, named 3′ cap independent translation enhancers (3′CITE) [85], were discovered in 1993 in a satellite tobacco necrosis virus [86].The 3′CITEs are able to interact with the initiation factor complex eIF4F, a component of the translation machinery, through their 3′-end structure (for a review of the structure of 3′CITE, see [87]). To assess the structure of the tomato bushy stunt virus (TBSV) 3′CITE, a combination of enzymatic (RNase S1 and V1) and chemical probing (DEPC and CMCT) was used and allowed to model the 5′-3′ interaction responsible for the delivery of the initiation factor to the 5′-end of the genome, through long-distance base-pairing [88]. The initiation factor recruits the 40S subunit of the ribosome, facilitating the translation of the viral RNA. Finally, even if the molecular mechanisms involved in enhanced translation differ from one 3′CITE to another; they remain interchangeable and fulfill a similar function [87].

##### RNA Structures of Human Pathogenic Viruses

The experiments performed with plant vRNAs paved the way for the chemical and enzymatic probing of other viruses. In this regard, a particular interest was expressed for human pathogenic viruses. One of them was poliovirus, a well-known member of the *Picornaviridae*. Indeed, structural elements involved in the recruitment of host ribosomes to promote translation of the viral proteins were discovered in the 5′-end region of picornaviruses [89,90]. One of the first internal ribosome entry site (IRES) secondary structure models to be built was the one from the encephalomyocarditis virus (EMCV), which was obtained through a combination of chemical (DMS) and enzymatic (RNase V1 and S1) probing [91]. Later, IRESes were found to be present in many viral families, with more or less complex structures able to promote translation initiation, usually in a cap-independent manner, without requiring the complete host translation machinery (for review, see [92]). Today, the structures and molecular mechanisms involved in the translation induced by IRES are extensively studied.

Moreover, picornaviruses structures were also studied to gain a better understanding of their virulence. In 1989, probing with kethoxal (along with DMS and RNases T1, T2, and V1) was used to identify the structural differences between a virulent and an attenuated poliovirus [65]. These data confirmed and completed a previous computer-predicted minimal energy structure [93] and provided additional evidence for the existence of an interaction in a region important for neurovirulence.

RNA viruses are not the only human pathogen relying on RNA structure for their infectivity. Indeed, the Epstein-Barr virus (EBV) (a *Herpesviridae*) codes for two small ncRNAs, EBER-1 and EBER-2, involved in latency and tumorigenic phenotypes. The structure of these two RNAs was studied using a combination of DMS, kethoxal, and CMCT to obtain information on all four nucleotides [69]. Associated with RNase V1 and nuclease S1, these reagents allowed the determination of the structure of EBER1 and EBER2 ncRNAs, either naked or in cell from extracted ribonucleoprotein (RNP) complexes. The modifications and cleavage sites were mapped by primer extension, except for the extreme 20 nucleotides at the 3′-end of the RNAs, for which enzymatic digestion and PAGE of the digests was necessary. Moreover, the authors determined the binding sites of the La protein, which constitutes the RNP complexes by immunoprecipitation with La-antigen followed by RNase T1 or RNase A digestion.

The appearance of the acquired immunodeficiency syndrome (AIDS) pandemic in the 1980s has led to a large number of probing studies of the human immunodeficiency virus type 1 (HIV-1) genomic RNA (gRNA) at different stages of the viral cycle and with a wide range of probes. For example, the structural domains, located in the 5′ terminal region, and their potential role in dimerization were studied by chemical probing with enzymatic and chemical probes [94,95]. *Neurospora crassa* endonuclease was used to study the tRNA^Lys,3^-gRNA reverse transcription initiation complex, together with a panel of chemical probes [67,96]. In addition to the predictable interaction between the viral primer binding site (PBS) and the 3′ extremity of the tRNA^Lys,3^, the authors were able to identify additional and specific contacts between an A-rich loop (located upstream of the PBS and the anticodon loop of tRNA^Lys,3^) by labeling the 3′ extremity of the tRNA and performing a denaturing PAGE on the RNA digests. To gain insight into the three-dimensional structure of the gRNA-tRNA^Lys,3^ complex, a complementary study with hydroxyl radicals was also performed [73]. In 1997, a variety of chemical probes, including lead ions, were used to study the influence of the purines flanking the dimerization initiation site (DIS) palindromic sequence on HIV-1 gRNA dimerization [76]. Pb^2+^ ions were used to detect subtle structural alterations between monomeric and dimeric forms of gRNA. The data allowed for the conclusion that the three flanking purines played major roles in the kinetics of RNA dimerization and in the stability of the dimers formed [76]. Later on, lead ions were also used to monitor the specific binding of aminoglycosides at the DIS in vitro and in cells [97]. Structural studies of the 5′-UTR (untranslated region) of HIV-1 gRNA were important for understanding the fate of this RNA between translation and packaging. Based on enzymatic and chemical probing, different models with alternative long-range interactions have been published, which could drive gRNA to the translation or packaging pathways [98,99,100].

##### Enzymatic Viral RNAs: Self-Cleaving Ribozymes

Many viral RNA structures are known to carry out important functions during replication. These functions often require the viral or/and cellular partners recruited by structural RNA motifs. However, some of the structures present in viruses, viroids, and virusoids are functional on their own once they have adopted a specific 3D structure [101]. This is the case for viral ribozymes, such as the hammerhead ribozymes, which are found in many ss circular RNAs and are responsible for the self-cleaving activity of the RNA during rolling circle replication. The first identified RNA with autocatalytic cleavage ability was the *Tetrahymena thermophila* IVS RNA [102]. In this study, the authors established that the secondary/tertiary structure of the RNA was involved in the cleaving activity. In 1989, this activity was also found in the hepatitis D virus (HDV) [103], and the secondary structures associated with the self-cleavage function in gRNA and antigenomic RNA were confirmed through enzymatic probing with RNases A, V1, T1, U2 [60], and [^32^P] 5′-end radiolabeled RNA. Since the discovery of these self-cleaving RNAs, they have been widely used as tools for controlling gene expression [104].

## 3. The SHAPE (r)Evolution

**Figure 2:**
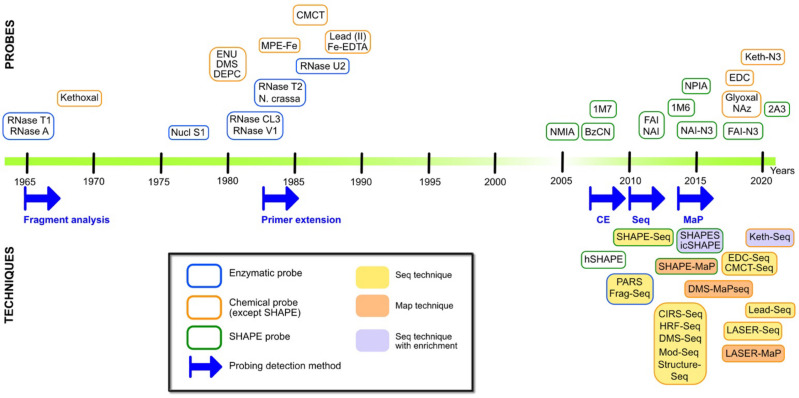
Timeline of RNA probes and probing techniques evolution. Upper part, enzymes are boxed in blue, chemical reagents (with the exception of SHAPE reagents) are boxed in orange, and SHAPE probes are boxed in green; the center of the box corresponds to the year of publication. Lower part, the blue arrows indicate the implementation of a new method to detect sites of cleavage or modification. CE: capillary electrophoresis; Seq: deep-sequencing; MaP: mutational profiling. Methods combining probing and deep-sequencing are highlighted in yellow. Methods based on mutational profiling are highlighted in orange, and techniques using enrichment by selection via the probe are labeled in purple. PARS: parallel analysis of RNA structure; FragSeq: fragmentation sequencing; CIRS-Seq: DMS and CMCT probing and sequencing; HRF-Seq: hydroxyl radical cleavage and sequencing; Mod-Seq: DMS probing and sequencing; Structure-Seq: DMS probing and sequencing; SHAPES: probing with NPIA and selection; icSHAPE: probing with NAI-N_3_ and selection; Keth-seq: N_3_-kethoxal probing, selection, and sequencing; LASER/-seq/-MaP: light-activated structural examination of RNA/analyzed by sequencing/mutational profiling.

Since the beginning of chemical probing, probes have been selected for their ability to highlight the exposure to the solvent, flexibility, or ss nature of the nucleotides. A combination of a few probes was usually required, in order to establish an experimentally validated secondary structure model of an RNA of interest. This changed in 2005, with the work of Merino et al. [105]. Indeed, in previous work on 2′-NH_2_-nucleotides, this laboratory had noticed that the 2′ position of nucleotides with a flexible sugar ring, which generally correspond to unpaired nucleotides, was more reactive than that of base-paired nucleotides [106]. Therefore, they designed a new chemical probe, the N-methylisatoic anhydride (NMIA), capable of acylating the 2′-hydroxyl group of nucleosides and tested its potential as a structural probe by studying the structure of yeast tRNA^Asp^ [105]. The resulting mapping method was named SHAPE for selective 2′-hydroxyl acylation analyzed by primer extension (Figure 1 and Figure 2).

The first notable improvement brought by SHAPE was that it allowed for the interrogation of the 4 nucleotides of RNA in a single experiment and, thus, to map all the reactivities of an RNA molecule using a single probe [105,107,108]. NMIA was only the first of a series of reagents that emerged in the following years (Table 2). First, introducing a nitro group in para of the electrophilic carbonyl, 1-methyl-7-nitroisatoic anhydride (1M7) was found to be much more reactive than NMIA (half live of 14 s vs. 10 min) [109,110] and the reaction kinetic was found to be even faster for the commercial reagent BzCN, which reacts on 2′OH in 0.25 s. [109]. While BzCN is well suited for the study of dynamic RNAs and transient RNA structures [111], other compounds were designed to be less reactive, in order to be compatible with the study of RNA structures. This is particularly the case for 2-methyl nicotinic acid imidazolide (NAI) and 2-methyl-3-furoic acid imidazolide (FAI) (t_1/2_ = 33 min and 73 min, respectively) [112,113].

**Figure 3:**
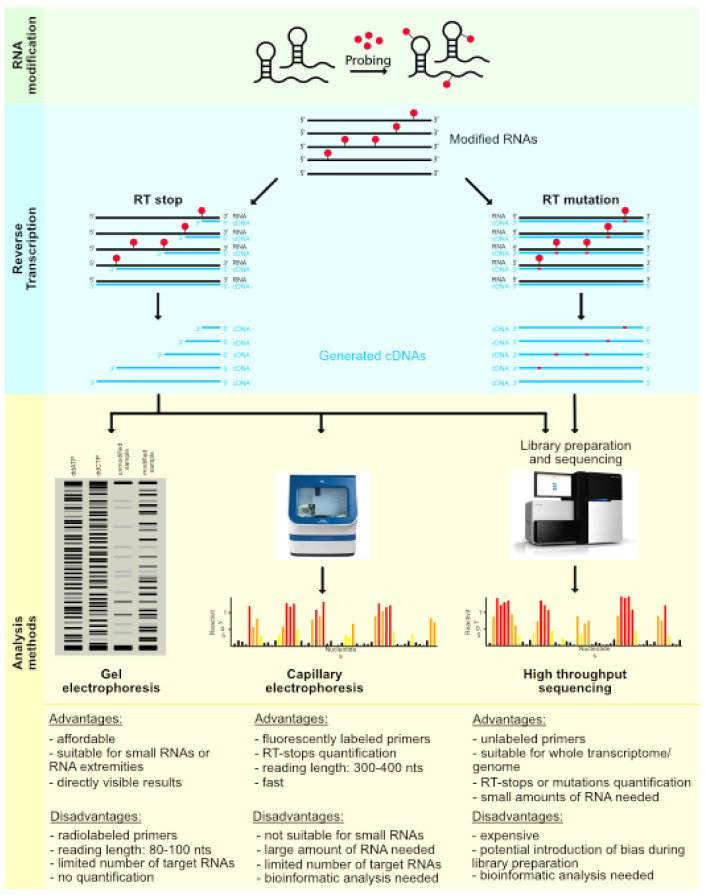
Schematic representation of RNA probing methods based on chemical modification. Folded RNAs were modified with the chemical probe of interest (green panel). For the RT stop strategy (left, blue panel), modified RNAs were reverse-transcribed with (1) radiolabeled primers for gel electrophoresis visualization (left, yellow panel), (2) fluorescently labeled primers for capillary electrophoresis detection (center, yellow panel), or (3) unlabeled primers for high-throughput sequencing after adapter ligation and library preparation (right, yellow panel). For the mutational profiling approach (right, blue panel), the modified RNAs were reverse-transcribed under mutation-inducing conditions with unlabeled primers. The cDNAs were ligated to adapters, and a library was prepared for high-throughput sequencing (right, yellow panel). The main advantages and disadvantages of the three analysis methods were featured at the bottom of the yellow panel.

Another important improvement concerns the coupling of SHAPE mapping with adduct detection by capillary electrophoresis (CE), in order to allow for high-throughput and quantitative analysis (hSHAPE). Indeed, the initial approach consisted of extending radioactive primers and visualizing the cDNAs obtained by PAGE, but the methodology quickly evolved with the use of fluorescently labeled primers. In that case, the cDNAs obtained after the reverse transcription step can be separated by capillary electrophoresis and quantified on DNA sequencers [109] (Figure 3).

The switch from PAGE to capillary electrophoresis has had multiple consequences. First of all, the reproducibility and resolution of the results have greatly improved, which has allowed for obtaining information for every nucleotide and to increase the length of the reading windows from approximatively 80–100 nucleotides to 300–400 nucleotides in a single experiment, making the study of large RNAs more accessible [114]. Second, the number of experiments to be analyzed in one working day (with a DNA sequencing analyzer instrument equipped with a capillary array) was also increased, allowing for the study of more RNAs and/or experimental conditions. Thirdly, the new format of the generated data is digital, similar to DNA sequencing traces, so they must be processed before being used. In addition, the analysis must be able to compare the different experiments; consequently, there was a need for bioinformatics tools to handle all these steps in efficient, automated, and accurate processes. Over the years, several software have been developed to solve this problem, such as CAFA [115], ShapeFinder [114], HiTRACE [116], FAST [117], QuShape [118], and RiboCAT [119]. Some tools have been developed in recent years to complement the analysis performed by the previously mentioned software, such as RNAthor [120] and RNAProbe [121].

SHAPE was successfully applied to the study of the RNA structures of several human pathogens. Using 1M7, Watts et al. [126] were able to interrogate 99.4% of the 9173 nucleotides of HIV-1 gRNA and to obtain a complete SHAPE-validated RNA secondary structure model. Poliovirus RNA was also studied with the NMIA probe [127] and hepatitis C virus with NAI [128].

Besides the hSHAPE technique, other “old probes” have also been coupled to CE detection, in order to gain more information on RNA structures. Actually, SHAPE probes interrogate the sugar face of the RNA, but information on the Watson–Crick face accessibility are also of interest; chemical probes, such as DMS [129,130,131] and CMCT [130,131], which interrogate the nucleobases, along with RNases [130], have successfully been applied to RNA structure determination in a way similar to hSHAPE.

## 4. A New Era for Enzymatic and Chemical Probing

### 4.1. The Deep-Sequencing Leaps

Even if hSHAPE and its further modifications constituted important advances in RNA structure determination by decreasing the workload of the experiments and facilitating quantification, the improved, but still limited, output and the large amount of RNA required for the detection of the modified nucleotides were the two biggest limitations of this technique. Indeed, for studies that are limited to small regions (and when the amount of RNA available is low), it is preferable to use the older method, consisting of the extension of radiolabeled primers, followed by analysis by PAGE, instead of the extension of fluorescent primers analyzed on a DNA sequencer. The coupling of RNA probing with next-generation sequencing (NGS) allowed the community to overcome these limitations and step into a new era of transcriptome- and genome-wide studies of RNA structure (structurome).

Structurome studies began in 2010 with enzymatic probes, and two methods were developed almost simultaneously: parallel analysis of RNA structure (PARS) [132] and fragmentation sequencing (Frag-Seq) [133]. PARS combined nuclease S1 and RNase V1 digestion, providing information on the ss- and ds RNA regions, respectively, followed by high-throughput sequencing of the dsDNA library, whereas Frag-Seq used only one enzyme (nuclease P1) but took two controls into account to gain information. Although the PARS method has been improved, notably in the form of PARTE [134,135] and nextPARS [136], methods involving enzyme probes have given way to chemical probe-based techniques. Indeed, soon after the development of PARS and Frag-seq, SHAPE probes were coupled to NGS (SHAPE-Seq [137] and SHAPE-Seq 2.0 [138]), since, unlike enzymes, SHAPE probes can be used in cells, as well as in a wider range of temperature and buffer conditions (Figure 2). The millions of reads generated by deep-sequencing make the SHAPE-Seq a more sensitive technique than hSHAPE. Furthermore, this method can be adapted to get transcriptome-wide structural information or by using sequence-specific reverse transcription primers to focus on the structure of an RNA of interest [12,139]. However, particular attention must be paid to the construction of the library because biases can be introduced at this level, especially during the ligation of adapters and/or during the PCR amplification step (Figure 3).

### 4.2. Old Probes Back in the Spotlight

Given the advances made possible by SHAPE-seq, “old” probes have also been revisited, in combination with deep-sequencing detection. A multitude of techniques have emerged that often vary only in one of the steps of the library preparation. HRF-Seq [140] takes advantage of hydroxyl radical cleavages; DMS was used in structure-Seq [141], DMS-Seq [142], Mod-Seq [143], and CIRS-seq [144]. This last technique actually combines DMS and CMCT to probe all four nucleotides. More recently, *N*-cyclohexyl *N*′-(2-morpholinoethyl) carbodiimide, the reactive group of CMCT, was also used in the Seq technique to map the pseudouridine positions in rRNA [145], as well as 1-ethyl-3-(3-dimethylaminopropyl)carbodiimide (EDC), a cell permeable derivative of CMCT [124,146] (Figure 2 and Table 2). In a similar way, lead(II) [147] has been coupled with NGS and is also experiencing a renewal. Since each probe possesses its own bias or specificity, increasing the number of probes available for studying RNA structures and RNA-protein interactions allows us to provide more pieces to the RNA and RNP structure puzzles.

The increasing evidence that G-quadruplexes (G4s) structures are important in the virus life cycle [148,149], and the fact that they may constitute a therapeutic target, has led to the application of the high-throughput mapping approaches for their study (for a review, see [150]). The previously established techniques, such as RT stop footprinting [151] and SHALiPE [150], have been improved to rG4-Seq [152], SHALiPE-Seq [153], or G4RP-Seq [154]. Another high-throughput based method, Keth-Seq [155], can also be applied to G4s studies, even if it is a non-rG4-specific method and it cannot differentiate G4s from dsGs.

Moreover, other improvements have been made to the “PROBE-Seq” techniques by introducing a second and orthogonal reactive group inside pre-existing probes. Using bi-functional probes, such as N-propanone isatoic anhydride (NPIA) in the SHAPES technique [122], NAI-N3 and FAI-N3 in icSHAPE [123] (which has been recently used to probe SARS-CoV-2 gRNA structure [156]), and Khetoxal-N3 in Keth-Seq [155], it was possible to link biotin to the modified RNA molecules via a click chemistry reaction. Selection with streptavidin beads allowed for the enrichment of the RNA of interest, thereby increasing the signal-to-noise ratio and significantly improving the sensitivity.

### 4.3. Mutational Profiling

Mutational profiling (MaP) was perhaps the most important improvement in determining the structure of RNAs by simplifying and reducing biases during library preparation. It was first adapted to SHAPE as SHAPE-MaP [157,158] and later, with the widely used DMS probe, as DMS-MaPseq [159]. These new techniques are reminiscent of whole-genome bisulfite sequencing (WGBS) [160], which has allowed the study of the human DNA methylomes by detecting mutations. These techniques use the capacity of reverse transcriptase to misread SHAPE or DMS-modified nucleotides and incorporate mutations in the newly synthetized cDNA. Whereas in the SHAPE-Seq and DMS-Seq strategies, modifications are identified as 3′-ends of the cDNA library, generated by stops during the reverse transcription; in the MaP strategies, mutations are detected all along the cDNA fragments. The generated cDNA library presents more homogeneity in size and can give more information, as it can contain multiple probe-induced mutations inside the same fragment (Figure 3). These features make it possible to get rid of the adapter ligation bias and to better identify the positions and frequencies of modification, even with very small amounts of RNA.

Recently, new chemical probes were designed and adapted to the MaP strategy (Figure 1 and Figure 2). Under light-activation, nicotinoyl-azide (NAz) generates nicotinoyl nitrenium ions that react with the C8 position of Gs and As, whether they are ss or ds. This probe was originally used in a “classical way” in the LASER (light activated structural examination of RNA) technique [125], to interrogate the RNA solvent accessibility and the ligand binding sites. However, NAz is compatible with deep-sequencing in LASER-Seq and LASER-MaP [161], as it induces RT-stops, as well as misreadings. A new SHAPE probe, compatible with mutational profiling, was also developed to improve in cellula studies: 2-aminopyridine-3-carboxylic acid imidazolide (2A3) [162].

### 4.4. The Latest Developments

Nevertheless, the probing techniques described above are still limited because, for example, they do not directly identify base-pairing interactions, rather they infer them from the compatibility that may exist between the observed reactivity and the proposed structure. That is why even more sophisticated methods have been developed. Among them, the “RNA interaction groups analyzed by mutational profiling” (RING-MaP) technique [163] allows for the identification of nucleotides that interact with each other. Indeed, when RNA is highly modified with DMS, these nucleotides are seen, after reverse transcription, as correlated patterns of mutations in the cDNAs [164]. Depending on how the correlation data are interpreted, it is possible with this technique to (1) detect RNA duplexes with the “pairing ascertained from interacting RNA strands measured by mutational profiling” (PAIR-MaP) method [164,165], (2) identify multiple RNA subpopulation structures present in the solution [163], and (3) reveal tertiary interactions [163,166,167]. This technique was further improved by replacing DMS with the highly reactive trimethyl oxonium (TMO), which allowed for the time-resolved probing of RNA [168]. In order to identify alternative conformations of RNAs, data from DMS-MaPseq experiments can also be analyzed by “detection of RNA folding ensembles using expectation-maximization” (DREEM) [169]. This algorithm allows the detection of heterogeneous regions inside the HIV-1 gRNA (and more precisely, at splice sites) that may explain the regulation of the gRNA packaging. {Citation}In another approach, merging mutate-and-map [170,171] with massively parallel DNA sequencing technology in M2-seq [172] generates helix signatures. Here, mutations are first introduced by error-prone PCR, and the effects of the mutations on nucleotide-pairing are detected by DMS probing.

Another interesting application of MaP is the localization of the protein interaction sites on RNAs by the RNP-MaP technique [173]. Indeed, with classical mapping methods, it is quite complicated to differentiate nucleotides that are protected by base pairing from those protected by the binding of a protein. In RNP-MaP, the cell permeable reagent NHS-diazirine reacts with the lysine of proteins, thanks to its succinimidyl ester function and with nucleic acids, after the activation of its diazirine moiety by long-wavelength UV. The RNA-protein crosslinks are then detected by mutational profiling reverse transcription, and the protein binding sites can be deduced in a more reliable way. This is a very interesting first implementation for footprint experiments, but the development of a set of probes, similar to what happened with the chemical mapping probes, would be a plus to extend this technique.

There is no doubt that these latest developments have greatly improved the study of RNA structures and will pave the way for more in-depth studies. However, performing such experiments, involving a high rate of RNA chemical modifications, may induce structural changes during the probing experiment, and careful controls must be carried out to avoid analysis bias.

### 4.5. Applications of the MAP Strategies to Viral RNAs

These powerful techniques were applied to the study of several viruses. In recent years, the global structure of the influenza A virus (IAV) genome was undertaken by SHAPE-MaP experiments, with a 1M7 probe in vitro and inside virions [174]. The authors were able to detect both intra- and inter-interactions between the ss viral RNA fragments. DMS-MaPseq was also used to better understand IAV infection, at that time by studying mRNA in infected living cells; Simon et al. [175] identified the stable structural domains important for IAV replicative capacity. The SHAPE-MaP technique with 1M7 has also been used to study the chikungunya virus (CHIKV) gRNA [176]. The high-throughput flow allowed for the identification of regions where the RNA secondary structure is conserved between CHIKV isolates; some of them, already identified, are known for their importance in the functionality of the virus.

Since 2020, the highly pathogenic coronavirus, severe acute respiratory syndrome coronavirus-2 (SARS-CoV-2), has also been studied with mutational profiling approaches. SHAPE-MaP [177,178] and DMS-MaPseq [179,180] have been used to study the full RNA genome in infected cells, and these techniques identified conserved ss segments, as well as folded structures that could constitute RNA drug targets. It is reasonable to think that in the coming years, the expansion of the MaP strategy to the other chemical probes will be realized for the study of RNA structuromes.

## 5. Conclusions

Research on enzymatic and chemical probes in the second half of the last century has led, in barely 20 years, to the creation of a toolbox of probes that complement each other and that have allowed for the identification of many RNAs structures, as well as ligand-binding sites. Quickly after their discovery, these techniques have successfully been applied to the study of viral RNAs and have enabled a better comprehension of the biological roles of their structural features. However, the notable shortcomings of these methods were their inability to quickly provide robust structural information, as the generation of data was slow and quantification of the experiments was difficult, complicating data analysis.

In 2005, these shortcomings were addressed with the development of a new class of chemical probes targeting the sugar 2′-OH, regardless of the nature of the base and, later, the use of fluorescent primers and analysis of the cDNAs by capillary electrophoresis. These advances gave a new impetus to the RNA structural probing field by allowing for large-scale analyses in a shorter time, with quantifiable results that were easier to reproduce and compare.

Most importantly, these steps in the direction of high-throughput probing have paved the way for even more performant techniques: the next-generation sequencing-based RNA structure probing technologies. The methods initially developed with enzymatic probes were abandoned over time, in favor of the more versatile chemical probes. Indeed, one after another, almost all the former chemical probes have been coupled to deep-sequencing, which provides a more diverse and adaptable array of tools, available for the study of many RNA species and contexts.

## 6. Perspectives

As the novel combinations of probes with various methods based on NGS technology become more common, new challenges will arise in the RNA structural probing field. Thanks to the decades of development of the processing algorithms, the RNA secondary structures can be predicted in silico with or without probing information, even if experimental data are required to sharpen and confirm the predicted models. This points toward an increasing need for tools, in order to address the 3D structures of RNA molecules, as many functions and mechanisms rely on long-range interactions and complex folding structures. Some steps have already been taken toward this goal, with the recent development of SHAPE-JuMP [181], but also with other methods, such as LIGR-seq [182], PARIS [183], COMRADE [184], and MOHCA-seq [185].

Besides, a new window is presently opening in the field of RNA probing with nanopore sequencing, which allows for the direct detection of the RNA modification on individual (even very long) molecules, without the need for a reverse transcription step. The coupling of nanopore sequencing with RNA sequencing should prove especially useful in the analysis of co-existing RNA structures, which is difficult with ensemble techniques [186].

Finally, it is also crucial to remember that the structural information in the RNA field is required to decipher the functions carried out by RNA molecules. In order to establish a clear link between RNA motifs and their molecular functions, new ultrahigh-throughput methods were developed, which showed promising results in predicting the impact on single elements of RNA functions [187].

## Figures and Tables

**Table 1:** Enzymatic and chemical probes from 1965 to 2005. Abbreviations are as follows: A: adenosine; G: guanosine; C: cytidine; U: uracil; N: any nucleotide; ssN: any unpaired nucleotide, regardless of the base; ssA (G, C, or U): unpaired adenosine (guanosine, cytidine, or uracil); dsN: any paired nucleotide; HDV: hepatitis D virus; TYMV: turnip yellow mosaic virus; HIV-1/2: human immunodeficiency virus type 1/2; EBV: Epstein–Barr virus; •OH: hydroxyl radical. * indicate the publications that concern nucleic acids.

Probe	Target	In Cell-In Viro	Original Publication (* Related to Nucleic Acid)	Early Use as Structural Probe	Application to Viral RNA
Enzymatic					
RNase A	ssC and U	No	Markham and Smith (1952) * [58]	tRNA^Ala^ (1965) [21]	HDV (1991) [59]
RNase T1	ssG	No	Sato and Egami (1957) * [22]	tRNA^Ala^ (1965) [21]	TYMV (1982) [60,61]
Nuclease S1	ssN	No	Harada and Dahlberg (1975) * [31]	5S rRNA (1977) [62]	Bromoviruses (1981) [63]
RNase CL3	ssC>>A>U	No	Levy and Karpetsky (1980) * [33]	TYMV RNA (1982) [60]	TYMV (1982) [60]
RNase V1	4–6 nts in helices or stacked nts	No	Favorova et al. (1981) * [38]	5S rRNA (1982) [64]	TYMV (1982) [60,61]
RNase T2	ssA>ssN	No	Sato and Egami (1957) * [17]	yeast tRNA (1984) [32]	Poliovirus (1989) [65]
N. crassa endonuclease	ssN	No	Linn and Lehman (1965) [30]	tRNA^Trp^ (1984) [66]	HIV-1 (1993) [67]
RNase U2	ssA>G>>C>U	No	Uchida et al. (1970) * [34]	16S rRNA (1987) [68]	HDV (1991) [59]
Chemical					
Kethoxal	N1 and N2- ssG	No	Staehelin (1959) * [41]	Yeast tRNA (1969) [42]	EBV (1988) [69]
DMS	N7-G/N1-ssA N3-ssC	Yes	Lawley and Brookes (1963) * [26]	Yeast tRNA^Phe^ (1980) [45]	TYMV (1982) [61]
DEPC	N7-A	No	Oberg (1970) * [43]	Yeast tRNA^Phe^ (1980) [45]	TYMV (1982) [61]
ENU	Phosphates of ssN and dsN	No	Singer (1976) * [49]	tRNA^Phe^ (1980) [50]	TYMV (1982) [60]
CMCT	N3-U and N1-G ss	No	Augusti and Brown (1965) * [28]	16S rRNA (1986) [46]	EBV (1988) [69]
MPE-Fe(II) (^•^OH source)	Phosphate/sugar backbone dsN	No	Hertzberg and Dervan (1982) * [70]	tRNA^Phe^ (1984) [52]	HIV-2 (2013) [71]
Fe-EDTA (^•^OH source)	Solvent accessibility	Yes	Tullius and Dombroski (1986) * [72]	pre-mRNA (1989) [51]	HIV-1 (1999) [73]
Lead (II)	Phosphate/sugar backbone ssN	Yes	Werner et al. (1976) * [74]	16S rRNA (1989) [75]	HIV-1 (1997) [76]

**Table 2:** SHAPE and post-SHAPE chemical probes.

Probe	Target	In Cell/In Viro Probing	Early Use as Structural Probe
SHAPE	2′-OH ssN	Yes except BzCN	[105,107,108,109,112,122,123]
EDC	N3-ssU and N1-ssG	Yes	[124]
Glyoxal	N1-N2-ssG N1-N6-ssA N3-N4-ssC	Yes	[40]
NAz	C8-A and C8-G	Yes	[125]

## Data Availability

Not applicable.
